# Evaluation of the safety and insecticidal efficacy of ivermectin-treated bird feed formulations in different avian species

**DOI:** 10.1186/s13071-026-07311-6

**Published:** 2026-03-27

**Authors:** Michelle J. Savran, Kathryn Coffin, Claire M. Stewart, Chilinh Nguyen, Catalina Puska, Molly E. Ring, Anna-Sophia Leon, Preston Schweiner, Tilman Peters, Jenna C. Randall, Jenny Buczek, Paula Lado, Emily N. Gallichotte, Brady Clapsaddle, Christopher M. Barker, Gregory D. Ebel, Brian D. Foy

**Affiliations:** 1 Center for Vector-Borne and Infectious Diseases, Department of Microbiology, Immunology and Pathology, Colorado State University, Fort Collins, CO USA; 2 College of Veterinary Medicine, University of Leipzig, Leipzig, Germany; 3 Department of Biomedical Sciences, Colorado State University, Fort Collins, CO USA; 4 TDA Research Inc., Wheat Ridge, CO USA; 5 Department of Pathology, Microbiology & Immunology, University of California, Davis, Davis, CA USA

**Keywords:** Ivermectin, Mosquito control, *Culex tarsalis*, West Nile virus, Wildlife self-medication, Avian reservoir host

## Abstract

**Background:**

West Nile virus (WNV) is maintained in an enzootic cycle between reservoir host birds and *Culex* (*Cx.*) spp. mosquitoes. This relationship presents a potential target for vector control strategies. Ivermectin (IVM), an endectocidal drug that selectively affects invertebrates while remaining safe at high concentrations in mammals and birds, can be delivered to *Culex tarsalis* via blood meals from birds fed IVM-treated bird feed. In this study, we evaluated the safety, efficacy, and utility of IVM-treated bird feed as a novel vector control strategy by assessing its impact on multiple bird species and mosquitoes.

**Methods:**

Mosquitoes were collected during peak WNV transmission season in Northern Colorado and DNA extracted from blood meals to determine host species. Chickens, pigeons, zebra finches, and house sparrows were fed different formulations of IVM-treated bird feed and observed for clinical signs, and their sera were fed to *Cx. tarsalis* mosquitoes to evaluate mosquitocidal efficacy. Feeding rates and IVM serum concentrations in birds were analyzed using unpaired t-tests and one-way ANOVA, and mosquito survivorship was analyzed using Kaplan-Meier curves and compared using paired log-rank tests. IVM serum concentration and mosquito survivorship were compared using Spearman correlation.

**Results:**

Speciation analyses conducted on blood meals from *Cx. tarsalis* collected during peak WNV transmission season in Northern Colorado determined that they feed primarily on songbird species that commonly visit bird feeders, with house sparrows representing the most frequent blood meal host. In laboratory experiments using multiple formulations and doses of IVM, chickens, pigeons, zebra finches, and house sparrows ate comparable amounts of IVM-treated bird feed compared to untreated feed, had similar weight gain, and exhibited no clinical signs of toxicity. Both colony-reared and locally captured *Cx. tarsalis* showed significant mortality after feeding on sera from IVM-treated birds compared to controls.

**Conclusions:**

These results suggest that targeting songbirds with IVM-treated bird feed should be safe for wildlife and may elicit high rates of IVM-induced mortality by reaching a large proportion of WNV vector mosquitoes via their proclivity for feeding on passerine birds.

**Graphical Abstract:**

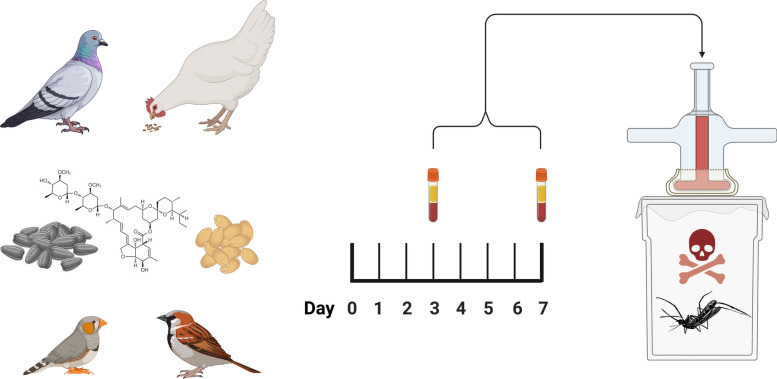

**Supplementary Information:**

The online version contains supplementary material available at 10.1186/s13071-026-07311-6.

## Background

West Nile virus (WNV) is the leading cause of locally acquired mosquito-borne disease in the USA, causing significant disease and death in humans and animals every year [1–3]. Since the introduction of WNV to the USA in 1999, over 56,000 WNV disease cases and over 2700 WNV-related deaths have been reported to the Center for Disease Control (CDC), with the highest incidence occurring in the western US and Great Plains region [4–6]. Moreover, models estimate the actual number of infections to be closer to 7 million, with approximately 1 million individuals developing disease [7]. While most infected individuals remain asymptomatic, about 20% develop febrile illness, and 1 in every 150 febrile cases develops neuroinflammatory conditions that often result in long-term disability or death [8, 9]. WNV has also impacted the health of North American bird populations since its emergence on the continent, inducing significant population declines in several species, which then result in increased susceptibility to other natural and anthropogenic impacts [3]. Additionally, more than 28,000 equine WNV cases have been reported to the US Department of Agriculture (USDA) with an average mortality rate of 30–40% [10]. Unfortunately, development and marketing of a WNV vaccine for human use remains unlikely in the foreseeable future because of the associated costs, epidemiological factors, and difficulties in planning and conducting clinical trials to prove its efficacy, leaving insecticides as the most practical strategy to control the spread of WNV [11, 12]. Insecticides primarily target the mosquito vector through biological and chemical interventions such as larvicidal bacteria, insect growth modulators, and adulticidal pyrethroid sprays [13–15]. Larvicides are critical for integrated mosquito management because of their low cost and high target specificity, but several predominant WNV vector species have developed resistance to the mechanisms of some of these agents and can be difficult to target in their larval habitats [16–18]. Adulticides can effectively reduce adult WNV vectors in treated areas throughout the USA, but adulticide applications can be expensive to implement, and success can vary by local infrastructure, thus limiting this option to wealthier urban and suburban communities [19–21]. Additionally, while numerous studies have demonstrated a strong safety profile when using the recommended application rates of the most often used adulticides, other studies have linked specific adulticides with toxicity for key pollinators, as well as threatened and endangered species, which has led to negative community perceptions and decreased utilization [22–25]. These drawbacks, combined with predicted future increases in WNV disease risk, indicate an urgent need for an alternative means of transmission control [26].

WNV is maintained in an enzootic cycle between passerine birds and ornithophilic *Culex* species mosquitoes. This ornithophilic blood-feeding habit can be used to target birds with novel interventions designed to control the spread of arboviruses like WNV [27, 28]. We have previously investigated the use of ivermectin (IVM)-treated bird feed to selectively target the main WNV bridge vector, *Culex tarsalis*, in the western USA [29–31]. IVM is an endectocide that targets the nerve and muscle cells of invertebrates, inducing flaccid paralysis and death within 2–3 days of exposure to the mosquito, while maintaining a robust safety profile in vertebrates [32–35]. Field research and modeling studies completed by some of our group suggest that IVM control strategies can also be used to control the spread of certain mosquito-borne pathogens such as malaria [36, 37]. IVM and other endectocides have been effectively employed for decades as both topical and feed-through treatments for many animals, from rodents to livestock, for tick, flea, and sand fly control [38, 39].

Due to their serial blood-feeding habits, mosquitoes would be expected to increase their exposure to IVM via avian blood meals as they age, so the presence of IVM-treated birds can increase the probability that WNV vectors will die before they reach an age where they could complete the extrinsic incubation period of the virus, decreasing the odds of transmission to another host [40]. Our previous studies have shown that *Cx. tarsalis* survival is significantly reduced following blood meals from chickens (*Gallus gallus*), wild Eurasian collared doves (*Streptopelia decaocto*), and a wild common grackle (*Quiscalus quiscula*) fed a simple formulation of IVM powder mixed into chicken feed (200 mg/kg feed) compared to controls [29]. In 2016, we placed IVM powder feed and control feed feeders in Fort Collins, Colorado. We observed doves and songbirds visiting all stations and detected IVM in sera of 13/15 (87%) of birds trapped around IVM-treated sites [29]. In 2019, we randomized eight flocks of chickens in Davis, California, and fed four flocks the same simple powder formulation for 72 days [30]. Despite no differences in mosquito abundance between flocks, we observed delayed and non-significantly decreased numbers of WNV-positive *Cx. tarsalis* pools at IVM sites relative to controls in 2016 and significantly reduced WNV seroconversion rates in chickens from IVM-treated flocks in 2019.

While our previous results indicate simple formulations of IVM-treated feed could impact WNV transmission in the field, there are outstanding gaps to address before pursuing field evaluation. We have observed birds at experimental feeder stations previously, but we need to confirm that mosquitoes will feed on bird species likely to visit the feeders. Additionally, the simple formulation we tested previously does not protect the drug from degradation and must therefore be replaced daily in the field, making it impractical. We have also not tested safety and efficacy for extended periods in small passerine birds. In this study, we assessed blood meal host preferences among mosquitoes captured in Northern Colorado to determine which species we should target with medicated feed for WNV control. We also tested new IVM-treated bird feed formulations to identify those that may work best in the field and simultaneously be safe for and induce mosquitocidal effects in multiple bird species.

## Methods

### Blood meal host analysis

Mosquitoes were collected and processed as described previously [41]. Briefly, CDC Miniature Light Trap Model 512 traps were set without light and baited with dry ice on volunteer properties throughout northern Colorado (Additional file 1: Table S1, host data listed in Additional file 1: Table S2). Trapped arthropods were brought back to CSU and frozen at −20 °C, and engorged *Cx. tarsalis* mosquitoes were identified. Engorged *Cx. tarsalis* mosquitoes were individually separated into 2-ml microcentrifuge tubes containing one sterile glass plating bead, 250 µl tissue lysis buffer, and 20 µl Proteinase K solution from the Mag-Bind® Blood & Tissue DNA HDQ 96 Kit (Qiagen, Valencia, CA). Mosquitoes were homogenized at 24 Hz for 1 min, centrifuged at 13,000×*g* for 5 min, and incubated at 55 °C in a shaking incubator overnight. DNA was extracted from mosquito homogenates via the Mag-Bind® Blood & Tissue DNA HDQ 96 Kit using a KingFisher™ Flex Purification System (ThermoFisher Scientific), according to the manufacturer’s instructions. Blood meals in extracted DNA were identified by polymerase chain reaction amplification and sequencing of a fragment of either the vertebrate mitochondrial cytochrome c oxidase 1 (COI) [42] or cytochrome b (cytb) [43, 44] genes, using the primer sets and thermocycling conditions described (Additional file 1: Table S3). Samples were submitted to Azenta Life Sciences for Sanger sequencing. The Barcode of Life Data System database was used to identify COI sequences (www.boldsystems.org), and GenBank was used to identify cytb sequences.

### Mosquito maintenance and bioassays

Colonized *Cx. tarsalis* (Kern National Wildlife Refuge strain) were reared in standard insectary conditions (28 °C, 16:8 light cycle). Approximately 150 larvae were reared in roughly 11 L of water and fed 2.5 g of powdered TetraMin fish food daily until pupation. Adults were housed in groups of approximately 300 per cage and fed sugar and water ad libitum until separated for bioassays. Field-collected *Cx. tarsalis* were captured in CDC Miniature Light Trap Model 512 traps (BioQuip) used without light and baited with dry ice. These were set throughout Fort Collins, Colorado (Additional file 1: Table S4) and brought back to CSU for holding. Trapped arthropods were knocked down by cold exposure, and *Cx. tarsalis* were identified, then separated into groups of 1:10 male:female and kept in standard insectary conditions (described above).

Prior to bioassays, mosquitoes were separated into groups of 50 females and 10 males, then starved of sugar and water for up to 18 h. For bioassays, mosquitoes were provided blood from birds either directly (fed on a live bird) or indirectly (artificial membrane feeder). As previously described for feeding laboratory mosquitoes bird blood, mosquitoes were fed indirectly by artificial membrane blood meals consisting of 50% avian serum and 50% red blood cells from defibrinated chicken or calf blood (Colorado Serum Company, CO, USA) via glass feeders (Lillie Glass Blowers, Smyrna, GA, USA) kept warm by a circulating water bath set to 37 °C [29]. Following 30 min of feeding, mosquitoes were knocked down by cold exposure and fully engorged females separated and mortality recorded daily for 7 days.

### Animals

Birds were provided with clean water and fed control or IVM-treated feed for 3 weeks (chickens, Fig. 2) or 7 days (all other birds, Figs. 3–6). Feed consumption was measured daily from heavy ceramic or cage-affixed bowls to prevent spillage (pigeons, zebra finches, house sparrows), and birds were weighed weekly (chickens), daily (pigeons), or every other day (house sparrows, zebra finches) to minimize stress. All birds were monitored daily for symptoms of toxicosis, including but not limited to mydriasis, abnormal stool, stupor, ptosis, and ataxia. Birds received a behavioral score of possible toxicity: (A) normal, no toxicity observed; (B) possible toxicity—with the action to monitor three times daily for signs of continued toxicity and for animals in the same group to have similar symptoms; (C) continued toxicity for >2 days, with the action that if noted as B for > 2 days, to be euthanized immediately; (D) > 2 birds per group develop continued toxicity, with the action that if > two birds per treatment group were noted as C, to stop the experiment with that dose and kileuthanize all animals in the group. All blood samples were allowed to clot for 30 min at room temperature, then separated into serum and red blood cells by centrifugation at 2000×*g* for 10 min. Serum was transferred to a 1.7-ml microcentrifuge tube and stored at − 80 °C until used for bioassays or IVM quantitation.

*Chickens*. Four-week-old white leghorn chickens (*Gallus gallus*) were purchased from Northern Colorado Feeders, divided into groups, and housed outdoors in separate chicken coops. All chickens were provided clean water and chick starter crumble mix (mash) from Northern Colorado Feeders Supply for 3 weeks, fed ad libitum. Birds in the treatment group received 200 mg of powder IVM formulation (Merck & Co., Inc., Kenilworth, NJ, USA) per kg of bird feed, mixed fresh daily. Blood was collected from each bird via jugular venipuncture once every 2 weeks. To compare mortality between field-collected and colonized *Cx. tarsalis* while controlling for variability in IVM serum concentration across time, chicken blood for artificial membrane feeds was drawn on the same day that field-collected mosquitoes were fed directly on the same chickens. Chickens were bled on an alternating schedule to evaluate mosquitocidal efficacy of serum over time without exceeding a biweekly maximum of 1% body weight blood collection.

*Pigeons*. Feral pigeons (rock doves, *Columba livia*) were captured in Larimer County, Colorado, and housed indoors in separate flight cages. All pigeons were provided clean water and either seeds, untreated IVM millet or sunflower seeds, or Opadry® II (Colorcon Inc., Harleysville, PA, USA)-coated IVM millet (TDA Research Inc, Golden, CO, USA). Blood was collected from each pigeon via brachial venipuncture on day 3 and day 7 of the diet.

*Zebra finches*. Zebra finches (*Taeniopygia guttata*) were purchased from a private breeder. Zebra finches were separated into groups (*n* = 3 for 50 mg/kg IVM diet, *n* = 8 for 100 mg/kg IVM diet) and provided clean water and millet diets, receiving control millet, untreated IVM millet, or Opadry II-coated IVM millet for 7 days. Blood was collected from each zebra finch after 7 days of the IVM diet via intracardiac puncture under terminal isoflurane anesthesia.

*House sparrows.* Wild house sparrows (*Passer domesticus*) were captured in Wellington, Colorado (40°42′57.8″N 105°01′09.9″W), divided into groups (*n* = 5 per each IVM diet, *n* = 3 per control diet), and housed indoors in separate flight cages. All sparrows were provided clean water and millet diets, receiving control millet, untreated IVM millet, or Opadry II-coated IVM millet for 7 days. Blood was collected from each house sparrow at the conclusion of the diet study via intracardiac puncture under terminal isoflurane anesthesia. Following euthanasia, house sparrow liver tissue was fixed in 10% neutral buffered formalin, trimmed and embedded in paraffin, sectioned at 5 µm, and stained with hematoxylin and eosin. Histopathological examination of liver tissue was performed by an anatomic pathologist who was blinded to treatment groups. The following lesional scoring system was used, and the summation of scores was compared across individuals and treatment groups: (1) minimal changes, (2) mild, (3) moderate, (4) marked, and (5) severe for each category (lipid vacuolization, hepatic nuclear toxic change, hepatic necrosis, and inflammatory cell infiltrate).

### IVM extraction and quantification by HPLC tandem mass spectrometry.

Serum (25 µl) was treated with 100 μl of 200 ng/ml abamectin (ABM) (Sigma-Aldrich, St. Louis, MO, USA) prepared in methanol (Honeywell, Charlotte, NC, USA) and vortexed until homogeneous. Samples were precipitated by freezing at −80 °C for 1 h and centrifuged at 30,000×*g* at 4 °C for 30 min. The supernatant layer was collected and evaporated under vacuum for approximately 1 h. The extract was reconstituted in 25 µl methanol, vortexed, and sonicated for 5 min. The resuspension was centrifuged for 30,000×*g* at 4 °C for 10 min; then, the supernatant was transferred to an HPLC vial with a screw cap.

Analyses were performed using an Agilent 1290 Infinity II liquid chromatograph system coupled to an Agilent 6470 triple quadrupole mass spectrometer electrospray ionization (ESI) source. The nitrogen dry gas flow rate was 10 l/min, held at 325 °C. The sheath gas flow rate was 11 l/min, held at 300 °C. Nebulizer gas pressure was 38 psi. The fragmentor voltage was 150 V. In positive polarity, the voltage capillary was 3500 V with a nozzle voltage of 0 V. In negative polarity, the voltage capillary was 3500 with a nozzle voltage of 2000 V. One quantifying and one qualifying MRM transition were monitored for both analyte and internal standard, each with a 150-V cycle time. For ivermectin, the precursor ion to product ion transitions were m/z 873.5 → 229.2 [quantifying, collision energy (CE) = 30 V] and m/z 567.5 (qualifying, CE = V). For abamectin, the precursor ion to product ion transitions were m/z 871.5 → 229.2 [quantifying, collision energy (CE) = 30 V] and m/z 565.5 (qualifying, CE = 30 V).

Reversed-phase separation of analytes was achieved on a Waters BEH C8 column (2.5 mm × 100 mm, 2.5 micron) using a gradient of 5 mM ammonium acetate in water (mobile phase A) and 5 mM ammonium acetate in 95% acetonitrile, 5% methanol (mobile phase B). The programmed gradient was as follows: hold 10% B from 0 to 1 min, linear increase to 99% B from 1 to 7 min, hold at 99% until 12 min, linear decrease to 10% from 12 to 12.5 min, and hold 10% B to 16.5 min. Chromatographic flow rate remained constant at 0.3 ml/min, and column temperature remained at 40 °C.

The linearity of the method was tested after elaboration of analytical calibration curves. For serum samples, nine concentrations (3.91, 7.81, 15.62, 31.25, 62.5, 125, 250, 500, 1000 ng/ml) of an IVM stock standard prepared in methanol were added to IVM-untreated chicken serum; 200 ng/ml ABM was added as the internal standard to assess extraction efficiency by comparing the peak areas of fortified blank samples with those of the added ABM. Standard curves were injected three times into the chromatography system to confirm accuracy and precision. The concentration of analyte present in a sample was then calculated following interpolation from the described calibration curve.

### Statistical analyses

Statistical analyses were performed using GraphPad Prism Version 10.6.0. Survival in mosquito bioassays was analyzed using Kaplan-Meier survival curves and compared using Mantel-Cox (log-rank) tests. Details regarding statistical tests comparing bird feed consumption rates and IVM sera concentrations are described in respective figure legends. IVM sera concentrations from birds were correlated to cumulative mosquito morality from bioassays conducted on respective animals using Spearman correlation.

## Results

**Fig. 1:**
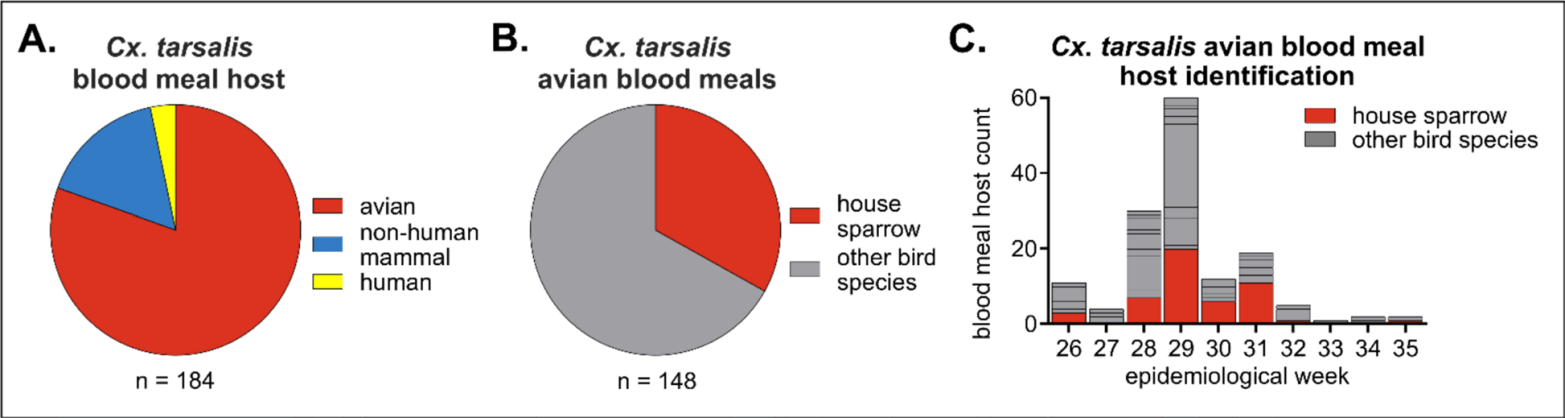
Blood meals identified from *Culex tarsalis* captured in Northern Colorado indicate house sparrows are a primary target. *Culex tarsalis* mosquitoes (*n* = 184) were trapped throughout Northern Colorado in summer 2023. **A** Blood meal host identification. **B** Avian blood meal host identification. All species and sample site details are listed in Additional file 1: Table S2. **C** Avian blood meal host identification throughout the WNV transmission season (epidemiological weeks 26–35).

To determine which species we could target in developing IVM-treated feed for field evaluation, we identified host blood meals from *Cx. tarsalis* collected from cities and towns located in the South Platte River valley in Northern Colorado during WNV transmission season (Additional file 1: Table S1). Most *Cx. tarsalis* blood meals came from avian hosts (84%) (Fig. 1A, Additional file 1: Table S2). Of these avian blood meals, 33% came from house sparrows, with each other detected avian species representing < 20% of identified blood meal hosts (Fig. 1B, Additional file 1: Table S2). Mosquitoes were fed on house sparrows throughout the WNV transmission season and were detected at 6/13 sample sites (Fig. 1C, Additional file 1: Table S2).

### Field-collected *Cx. tarsalis* are susceptible to IVM via avian blood meals

**Fig. 2:**
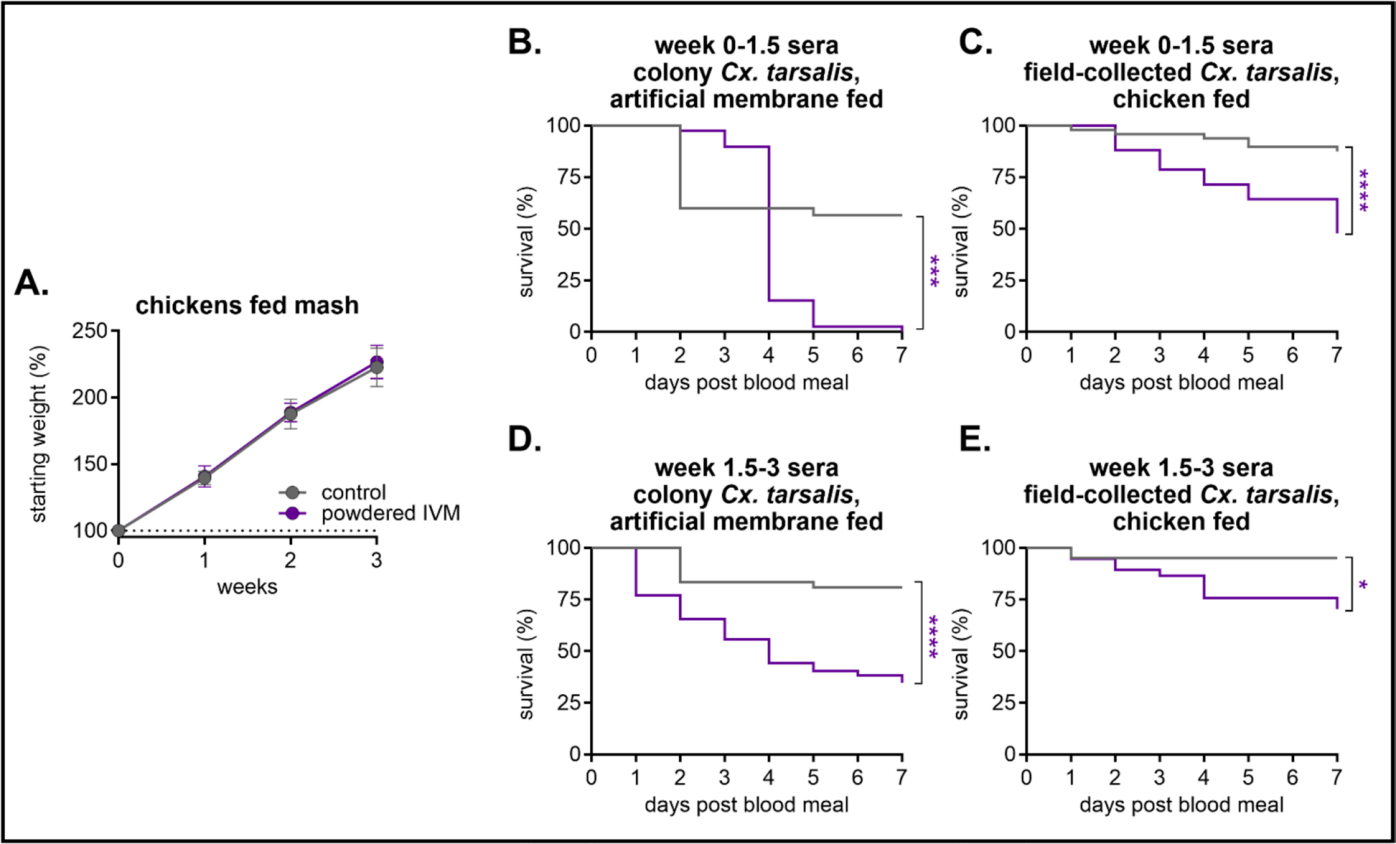
Field-collected *Culex tarsalis* mosquitoes experience significant mortality when fed chicken blood containing IVM. Chickens (*n* = 6 in control group, *n* = 5 in IVM-treated group) were fed control or 200 mg/kg of powdered IVM-treated mash for 3 weeks. **A** Chickens were weighed weekly (mean ± SD). **B**, **D** Colony and **C**, **E** field-collected *Cx. tarsalis* were fed **B**, **D** indirectly via an artificial membrane feeder or **C**, **D** directly on chickens throughout the course of the birds’ diet, and mortality was measured over 7 days. Log-rank (Mantel-Cox) test, *P < 0.05, ***P < 0.001, ****P < 0.0001

We previously determined consumption of 200 mg/kg of IVM-treated feed did not result in any adverse events in chickens and caused their sera to be mosquitocidal to colony-raised *Cx. tarsalis* mosquitoes [29]. To validate the efficacy of this formulation and dose in field-collected mosquitoes, we captured adult *Cx. tarsalis* in Larimer County, Colorado, from July through August 2021 (Additional file 1: Table S4). Four-week-old chickens (*Gallus gallus*) were exclusively fed 200 mg/kg powdered IVM in mash for 3 consecutive weeks, and both field-collected and colony-raised mosquitoes were fed blood meals derived from these birds throughout the diet period. All chickens remained healthy and exhibited no clinical symptoms with all scores reported as “A” per our behavioral protocol, and weight gain was similar between IVM and control feed groups (Fig. 2A). Consistent with our previous studies, colonized *Cx. tarsalis* experienced significant mortality after feeding on blood drawn from birds fed IVM-treated feed for up to 1.5 weeks, with 97% mortality after 7 days post-blood feeding [*n* = 30 mosquitoes fed on control sera, *n* = 39 mosquitoes fed on IVM-treated sera, Mantel-Haenszel hazard ratio = 3.449, 95% confidence interval (CI) = 1.661–7.162] (Fig. 2B). Similarly, field-collected *Cx. tarsalis* directly fed on IVM-treated chickens within the first 1.5 weeks of their diet experienced significant mortality, though they had lower mortality overall (52%) (*n* = 48 mosquitoes fed on control sera, *n* = 42 mosquitoes fed on IVM-treated sera, Mantel-Haenszel hazard ratio = 4.911, 95% CI = 2.261–10.67) (Fig. 2C). Mortality was overall less pronounced in both colonized and field-collected *Cx. tarsalis* fed blood from chickens consuming IVM-treated feed for up to 3 weeks, with 62% mortality in colonized mosquitoes (*n* = 42 mosquitoes fed on control sera, *n* = 52 mosquitoes fed on IVM-treated sera, Mantel-Haenszel hazard ratio = 4.273, 95% CI = 2.248–8.121) (Fig. 2D) and 24% mortality in field-collected mosquitoes (*n* = 20 mosquitoes fed on control sera, *n* = 37 mosquitoes fed on IVM-treated sera, Mantel-Haenszel hazard ratio = 3.569, 95% CI = 1.080–11.80) (Fig. 2E). Because trends were similar between field-collected and laboratory mosquitoes, and due to the challenge in collecting field mosquitoes during a limited time of year to feed them directly on live birds, we conducted all subsequent bioassays in colonized *Cx. tarsalis* mosquitoes.

### High-dose IVM-treated feed had a strong safety profile in pigeons and was mosquitocidal to *Cx. tarsalis*

**Fig. 3:**
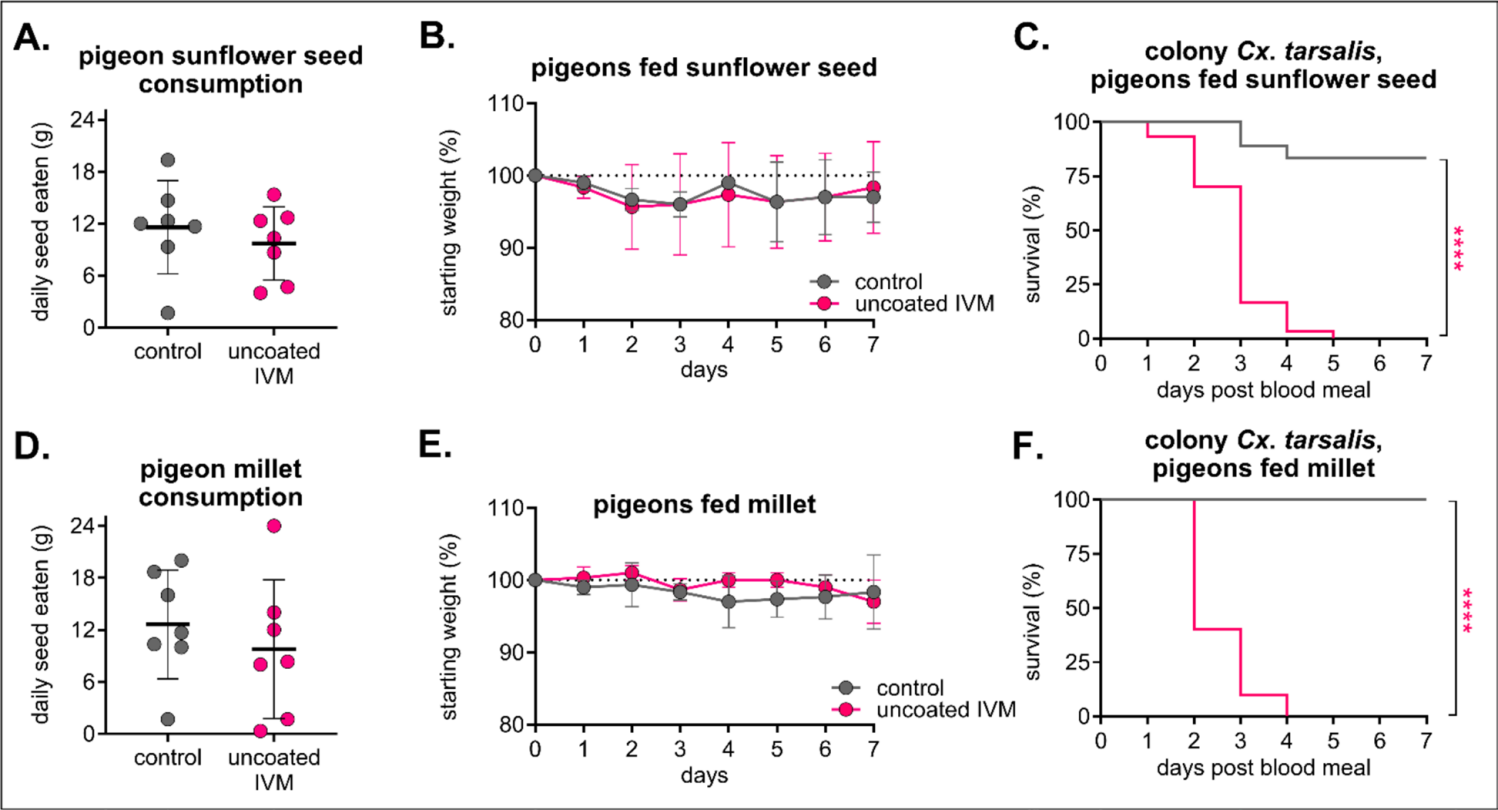
High-dose IVM-treated sunflower seeds and millet were well tolerated in pigeons and confer significant mortality to *Culex tarsalis*. Pigeons (*n* = 3 per group) were fed either control or 400 mg/kg uncoated IVM-treated **A–C** sunflower seeds or **D–F** millet for 7 days. **A, D** Total seeds eaten per group was measured daily (mean ± SD). Each point represents seeds eaten by group over the course of 1 day. No significant differences (*p* > 0.05) were observed between groups using an unpaired t-test. **B, E** Pigeons were weighed daily (mean ± SD). **C, F** Colony *Cx. tarsalis* were fed pigeon sera collected on day 3 after feeding on IVM-treated **C** sunflower seeds or **F** millet via an artificial membrane feeder, and mortality was measured over 7 days. Log-rank (Mantel-Cox) test, ****P < 0.0001

After observing less mortality in field-captured than in colonized *Cx. tarsalis*, we formulated a high-dose IVM diet (400 mg/kg) by spraying IVM onto commonly used seed diets (uncoated IVM) to increase mosquitocidal efficacy in a field setting. Sunflower seeds and millet were chosen to target a wide range of species likely to visit local bird feeders [45]. Domestic pigeons (*Columba livia*) were used to evaluate safety and efficacy in a species likely to consume seed from a bird feeder. Pigeons in all groups appeared healthy throughout and after eating the formulated diet, with similar rates of seed consumption (Fig. 3A/D) and weight change (Fig. 3B/E) and clinical scores reported as “A” as per our behavioral protocol. Colonized *Cx. tarsalis* experienced significant mortality after feeding on sera from IVM-treated pigeons compared to control, with 100% mortality after 7 days post-blood-feed in both IVM-treated sunflower seed (*n* = 18 mosquitoes fed on control sera, *n* = 30 fed on IVM sera, Mantel-Haenszel hazard ratio = 13.8, 95% CI = 5.823–32.71) and millet (*n* = 11 mosquitoes fed on control sera, *n* = 10 mosquitoes fed on IVM sera, Mantel-Haenszel hazard ratio = 39.58, 95% CI = 8.81–177.8) groups (Fig. 3C/F).

**Fig. 4:**
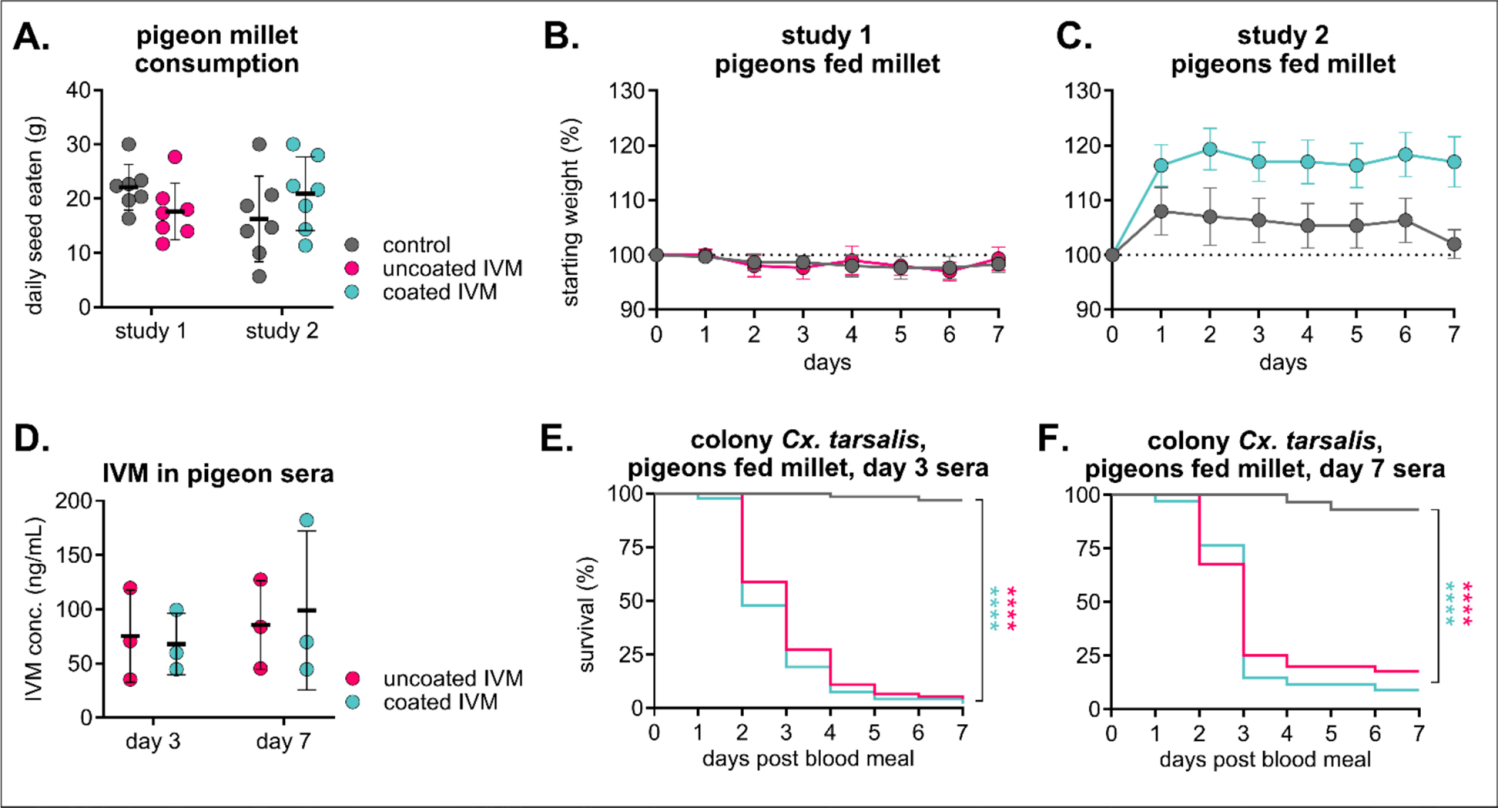
Excipient-formulated millet diets are palatable and efficacious. Pigeons (*n* = 3 per group) were fed either control or 200 mg/kg IVM-treated uncoated (study 1) or Opadry II-coated (study 2) millet for 7 days. **A** Total seed eaten per group was measured daily (mean ± SD). Each point represents seed eaten by group over the course of 1 day. No significant differences (*p* > 0.05) between groups within study using a two-way ANOVA were observed. **B–C** Pigeons were weighed daily (mean ± SD). **D** IVM was measured in pigeon sera, and there were no statistical differences within time points (*p* > 0.05) between IVM groups using a two-way ANOVA with with Šídák’s multiple comparison test. **E–F** Colony *Culex tarsalis* were fed pigeon sera collected on **E** day 3 or **F** day 7 via an artificial membrane feeder, and mortality was measured over 7 days. Log-rank (Mantel-Cox) test, ****P < 0.0001

To improve the environmental stability and pharmacokinetic properties of the diet, we developed excipient-formulated IVM-treated seeds coated with Opadry® II, a polyvinyl alcohol polymer commonly used in controlled-release formulations and tablet coatings. Because we observed complete mortality in mosquitoes fed sera from high-dose IVM-treated birds (Fig. 3), we selected the lower-dose formulation for evaluation in the following experiments. Pigeons consumed uncoated and coated IVM millet at similar rates compared to control feed (Fig. 4A), and all birds were considered healthy throughout the study, with similar weight change in both IVM groups compared to their respective controls and clinical scores reported as “A” as per our behavioral protocol (Fig. 4B/C). Additionally, IVM concentrations in pigeon sera were similar within matched timepoints between the uncoated and coated IVM groups (Fig. 4D). Colonized *Cx. tarsalis* fed day 3-collected sera from both IVM-treated groups experienced significant mortality, with > 95% mortality in both IVM groups compared to 3% in controls (*n* = 67 mosquitoes fed on control sera, *n* = 92 mosquitoes fed on uncoated IVM sera, Mantel-Haenszel hazard ratio = 20.45, 95% CI = 12.51–33.44; *n* = 94 mosquitoes fed on coated IVM sera, Mantel-Haenszel hazard ratio = 23.69, 95% CI 14.45–38.83) (Fig. 4E). The same effect was observed in *Cx. tarsalis* fed day 7-collected sera, albeit with comparatively higher survivorship rates of 83% mortality in uncoated and 91% mortality in coated compared to 7% in controls (*n* = 29 mosquitoes fed on control sera, *n* = 40 mosquitoes fed on uncoated IVM sera, Mantel-Haenszel hazard ratio = 11.77, 95% CI = 5.458–25.37; n = 34 mosquitoes fed on coated IVM sera, Mantel-Haenszel hazard ratio = 18.42, 95% CI = 8.036–42.24) (Fig. 4F). We did not observe any differences in mortality between mosquitoes fed blood collected on day 3 vs. day 7 (uncoated millet Mantel-Haenszel hazard ratio = 1.529, 95% CI = 0.9136–2.560, coated millet uncoated millet Mantel-Haenszel hazard ratio = 1.709, 95% CI = 0.9812–2.976) (Fig. S1).

### Excipient-formulated IVM-treated feed is well tolerated and effective in small passerine species

**Fig. 5:**
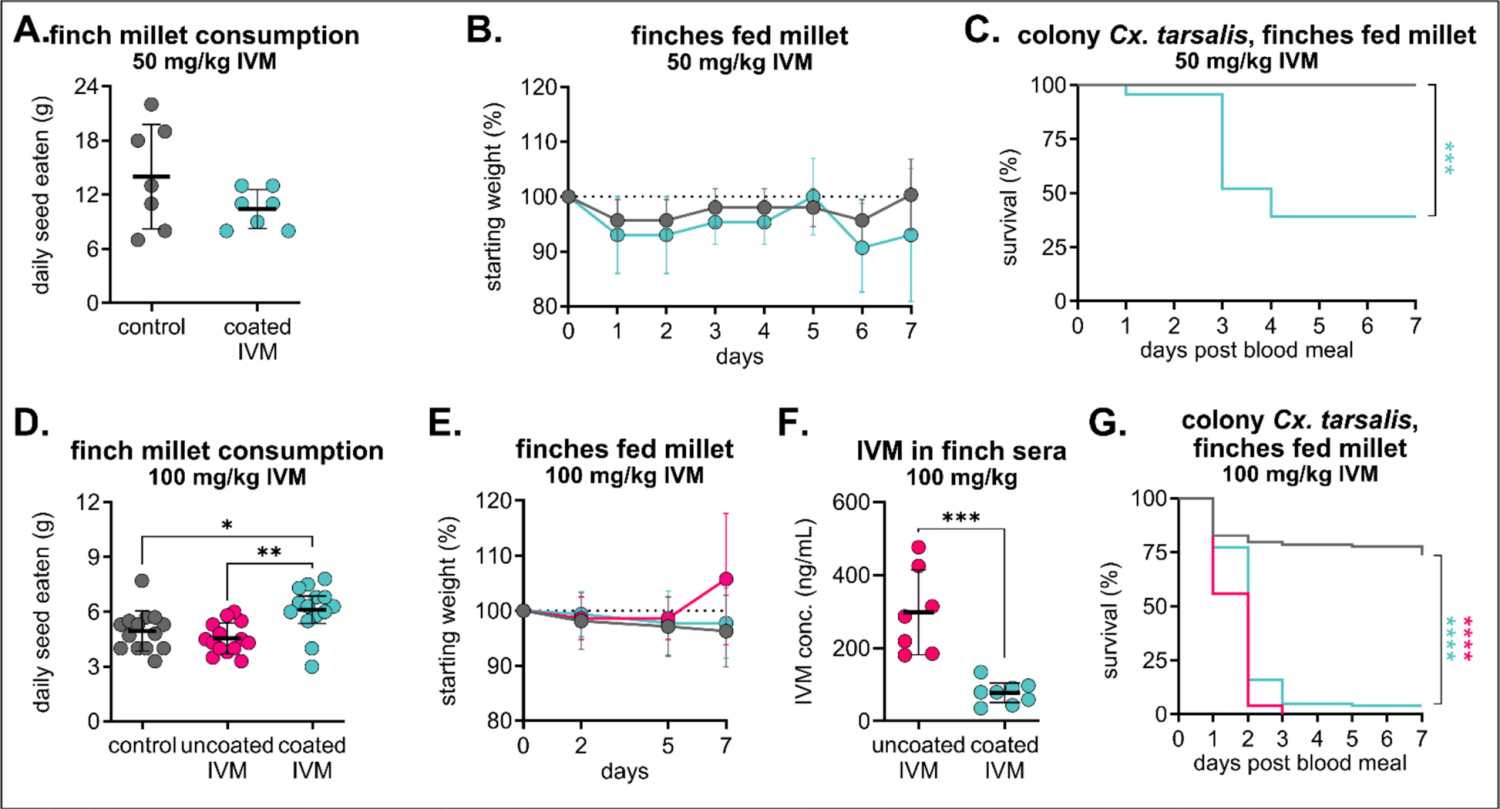
Mosquitocidal efficacy in zebra finch sera varies between doses and excipient formulations. Zebra finches were fed either **A–C** control or 50 mg/kg IVM-treated Opadry II-coated millet (*n* = 3 per group) or **D–G** control or 100 mg/kg IVM-treated uncoated or Opadry II-coated millet (*n* = 8 per each IVM group, 7 per control group) for 7 days. **A–D** Total seed eaten per group was measured daily (mean ± SD). Each point represents seed eaten by group over the course of 1 day. **A** No significant differences (*P* > 0.05) between groups were observed using an unpaired t-test. **D** One-way ANOVA with Tukey’s multiple comparisons test (*p < 0.05, **p < 0.005). **B, E** Zebra finches were weighed **B** daily or **E** four times over the 7-day diet period (mean ± SD). **C** Colony *Culex tarsalis* were fed zebra finch sera via an artificial membrane feeder, and mortality was measured over 7 days. Log-rank (Mantel-Cox) test, ***P < 0.005. **F** IVM was measured in sera of zebra finches fed 100 mg/kg uncoated and coated IVM millet diets. Concentrations were compared using an unpaired *t*-test (***P < 0.0005) **G** Colony *Cx. tarsalis* were fed zebra finch sera via an artificial membrane feeder, and mortality was measured over 7 days. Log-rank (Mantel-Cox) test, ****P < 0.0001

We next tested the same formulation (200 mg/kg IVM-treated feed) in zebra finches (*Taeniopygia guttata*) because of their small size, well-documented husbandry, and accessibility, establishing it as a valuable animal model to evaluate the approximate effects of this diet on passerine birds [46]. All zebra finches fed 200 mg/kg IVM-treated feed exhibited severe stupor, lethargy, and ataxia within 24 h of diet provision. After they had been checked by the laboratory animal veterinarian, birds were removed from diets, and all recovered within 24 h of withdrawal. After an additional 2-week withdrawal period to clear residual IVM, they were given a decreased concentration of 50 mg/kg and monitored three times daily. At this concentration, we did not observe adverse clinical signs throughout the duration of the diet, birds consumed experimental and control seed at similar rates, all scores were reported as “A” per our behavioral protocol, and all birds maintained their weight (Fig. 5A/B). While still significant compared to mosquitoes fed control sera, we observed less mosquito mortality (61% mortality) in those fed sera from coated IVM-treated birds (*n* = 12 mosquitoes fed on control sera, *n* = 23 mosquitoes fed on coated IVM sera, Mantel-Haenszel hazard ratio = 8.081, confidence interval 2.380–27.44) (Fig. 5C). We then increased the dose to 100 mg/kg IVM, and at this dose, even though birds in the coated IVM millet group consumed more feed than birds in the uncoated IVM millet group (Fig. 5D), all zebra finches remained healthy and maintained their weight (Fig. 5E). Interestingly, IVM concentrations in sera collected on day 7 from zebra finches fed uncoated IVM millet were significantly higher than those of coated IVM millet despite increased consumption in the coated IVM millet group (Fig. 5F). Despite these differences, colonized mosquitoes fed sera from both uncoated and coated IVM groups experienced comparable significant mortality, with 100% mortality in uncoated IVM and 96% mortality in coated IVM compared to 27% in controls (*n* = 98 mosquitoes fed on control sera, *n* = 77 mosquitoes fed on uncoated IVM sera, Mantel-Haenszel hazard ratio = 12.77, confidence interval 7.764–21.01; *n* = 101 mosquitoes fed on coated IVM sera, Mantel-Haenszel hazard ratio = 8.835, confidence interval 5.667–13.77) (Fig. 5G).

**Fig. 6:**
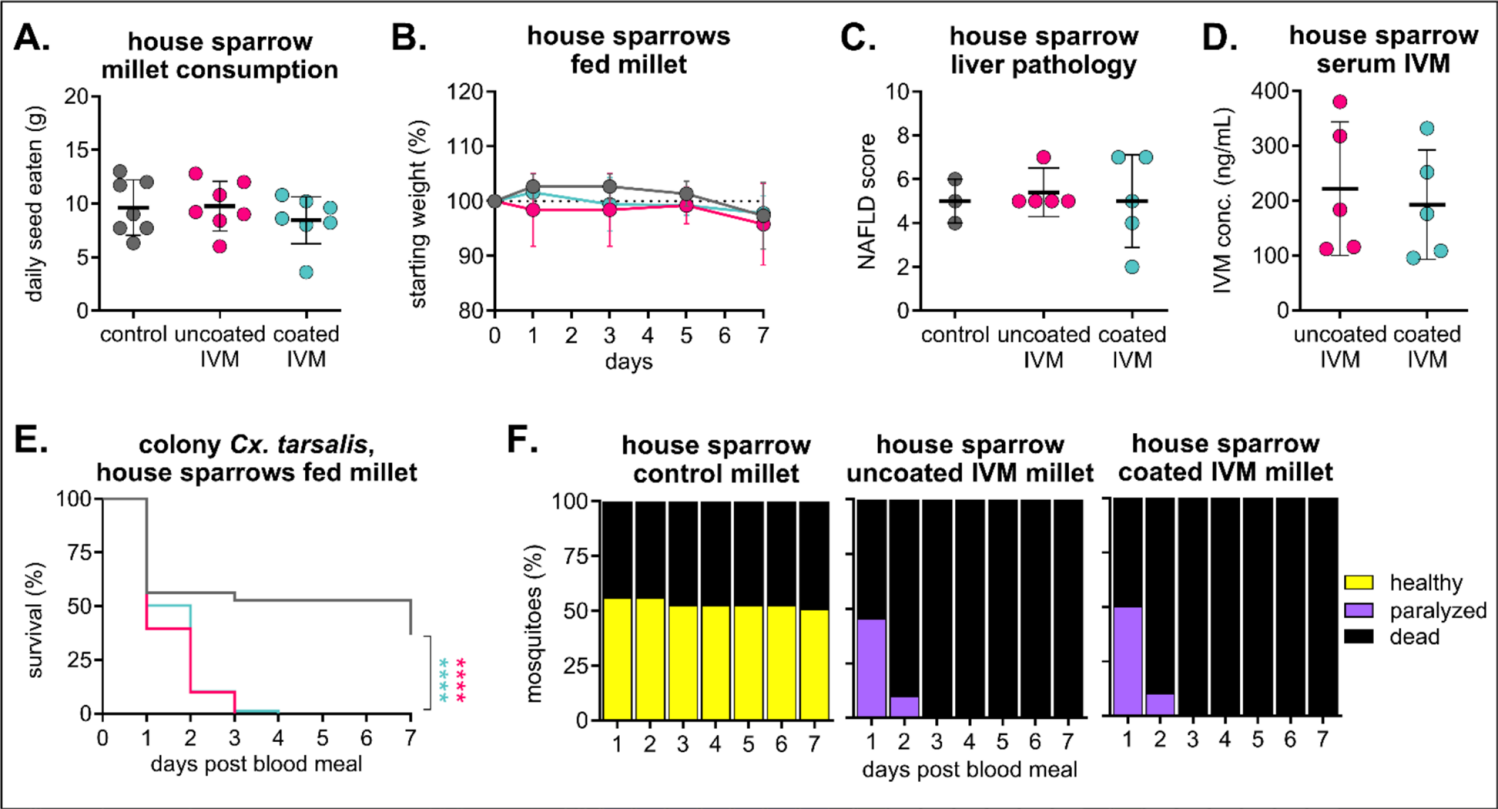
House sparrows safely consume IVM-treated feed and develop mosquitocidal concentrations in sera. House sparrows (*n* = 3 in control group, *n* = 5 per each IVM group) were fed 100 mg/kg IVM uncoated or Opadry II-coated millet for 7 days. **A** Total seed eaten per group was measured daily (mean ± SD). No significant difference (*p* > 0.05) between all groups using one-way ANOVA with Tukey’s multiple comparisons test. **B** House sparrows were weighed every other day (mean ± SD). **C** House sparrow liver tissue was evaluated and scored according to standards for non-alcoholic fatty liver disease (NAFLD). No significant difference (*p* > 0.05) was observed between groups using one-way ANOVA. **D** IVM was measured in house sparrow sera collected on day 7 of the diet. No significant difference (*p* > 0.05) between groups using unpaired t-test. **E, F** Colony *Culex tarsalis* were fed house sparrow sera via an artificial membrane feeder, and **E** mortality measured over 7 days. Log-rank (Mantel-Cox) test, ****p < 0.0001. **F** Blood-fed mosquitoes were observed for signs of toxicity for 7 days.

After determining that 100 mg/kg IVM feed was safe and efficacious in zebra finches, we evaluated the same dose and formulation in field-caught house sparrows (*Passer domesticus*). We selected house sparrows because they are a primary blood meal source for *Cx. tarsalis* mosquitoes in northern Colorado [41] (Fig. 1) and are frequently spotted at bird feeders [45]. All birds remained apparently healthy throughout the diet, and no differences in seed consumption or weight change were observed (Fig. 6A/B). Evaluation of liver histology revealed mild-to-moderate chronic hepatitis in all birds, including controls, and an overall lack of overt toxic changes in the treated group (Fig. 6C, Table S5). Unlike zebra finches (Fig. 5D), IVM concentrations in sera from house sparrows were comparable between the uncoated and coated IVM millet groups (Fig. 6D). Colonized mosquitoes fed sera from both uncoated and coated IVM groups experienced comparable significant mortality, with 100% mortality in both uncoated and coated IVM groups compared to 37% in the control (*n* = 57 mosquitoes fed on control sera, *n* = 160 mosquitoes fed on uncoated IVM sera, Mantel-Haenszel hazard ratio = 6.025, confidence interval 3.811–9.526; *n* = 165 mosquitoes fed on coated IVM sera, Mantel-Haenszel hazard ratio = 5.334, confidence interval 3.403–8.363) (Fig. 6E). A large proportion of mosquitoes fed sera from the control group experienced die-off 1 day post-blood-feeding, but remaining mosquitoes showed no signs of toxicity compared to mosquitoes fed sera from either IVM group, which were all paralyzed (Fig. 6F).

### Relationship among IVM formulation, real dose, and mosquitocidal efficacy

**Fig. 7:**
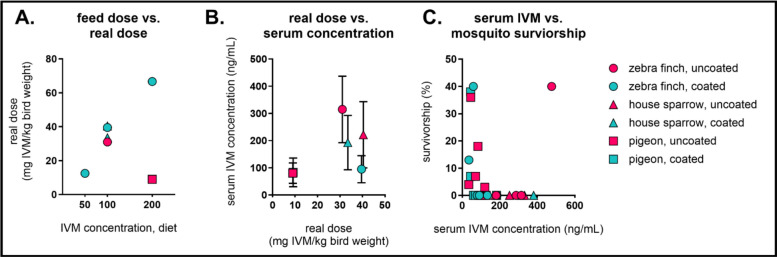
Relationships among IVM formulation, real dose, and mosquitocidal efficacy. Comparisons among real dose, IVM concentration in diet, IVM concentration in sera, and mosquito survivorship. **A** IVM dose in diet compared to real dose per average weight of birds. Real dose is calculated as follows: (kg of feed eaten per group × mg of IVM in diet)/(average weight of birds in group in kg). **B** Real dose per average weight of birds compared to IVM concentration in bird sera. **C** Individual avian IVM serum concentrations versus corresponding cumulative *Culex tarsalis* survivorship on day 7 post blood feeding.

To better understand the relationship between IVM formulation and dose, we extrapolated the real dose by calculating the total consumed IVM per bird weight and compared this value to the IVM concentration in the diet and in the sera from pigeons fed 200 mg/kg formulations, all house sparrows, and all zebra finches (Fig. 7A/B, Additional file 1: Table S6). The real dose ranged between 9 and 40 mg IVM/kg bird weight in all birds that appeared clinically healthy throughout the diet. Zebra finches fed the 200 mg/kg diet, which were the only animals to present signs of IVM toxicity following diet ingestion, received a real dose of 67 mg IVM/kg bird weight, substantially higher than for the other birds. Furthermore, we correlated the IVM concentration in individual birds’ sera to *Cx. tarsalis* survivorship (Fig. 7C, Additional file 1: Table S7). There were no observable relationships among species, formulation, and survivorship (Additional file 1: Table S8). Overall, *Cx. tarsalis* survivorship was < 50% in all groups irrespective of the real doses or IVM concentrations in sera and ≤ 40% in bioassays conducted with serum from passerine birds.

## Discussion

We report here that IVM-treated bird feed is likely to reach the preferred blood meal source of WNV vector *Cx. tarsalis* mosquitoes, has a strong safety profile for many avian species consuming it, and confers mosquitocidal efficacy to treated-bird serum when fed to both colonized and field-collected *Cx. tarsalis*. Birds as small as 11 g can tolerate eating exclusively 100 mg IVM per kg of bird feed for up to 1 week with no evidence of adverse clinical signs or histopathology. Colonized *Cx. tarsalis* are susceptible to serum from passerine birds fed formulated diets at 100 mg/kg, suggesting that this strategy can be effective at reducing WNV transmission in the field by targeting blood-feeding mosquitoes that prefer to feed on birds. Together, these data indicate that IVM-treated bird feed may be an effective WNV transmission control strategy via targeting blood-feeding adult mosquitoes when implemented in areas of high WNV transmission between songbirds and ornithophilic mosquitoes.

*Culex tarsalis* feeding behavior, as identified by blood meal analysis presented in this study, largely corroborated findings from previously published studies conducted in Colorado and other western states [41, 47], with most blood meals derived from avian hosts. A large proportion of blood meals came from common visitors to bird feeders, including house sparrows, mourning doves, house finches, and black-capped chickadees. We previously assessed the spatial movement of common birdfeeder species relative to bird feeder sites to determine how optimal deployment of IVM-treated feeders could reduce local transmission throughout the WNV season by reducing enzootic transmission prior to the onset of human infections [31]. In that study, we tagged house finches in Fort Collins, Colorado, with passive integrated transponders to quantify their visits to bird feeders [31]. Of the 2082 bird feeder visits, the average duration was ~21 s, and an individual bird was detected visiting a feeder 19 times per day. These data suggest small passerines will frequent the feeders often each day in the summer, coinciding with peak *Cx. tarsalis* activity. The success of an IVM-treated bird feed strategy for reducing virus transmission depends on *Cx. tarsalis* taking blood meals from self-medicated birds, so these data together with our blood meal host identification results suggest that mosquitoes are likely to take blood meals from house sparrows and other passerine birds that frequently visit bird feeders. All mosquitoes in this study were collected in CDC light traps baited with CO_2_, which attract host-seeking female mosquitoes. This dataset does not include mosquitoes captured in resting and gravid traps, which could bias blood meal distributions [48]. However, it is well documented that *Cx. tarsalis* primarily feed on birds and opportunistically feed on mammals, and our findings show the same overall distribution.

We developed IVM-seed formulations that are attractive to a broad variety of birds and can modulate drug release upon ingestion. Using both hulled millet and hulled sunflower seeds enables preferential targeting of both small and medium-sized granivorous birds, and we found comparable rates of consumption between treated and untreated seed within seed type and species groups. For IVM to be successful in the field, it also needs to be stable on a feed product. We also evaluated IVM-seed formulations coated with the aqueous excipient Opadry® II to maintain the stability of the active ingredient on the seeds and to modulate the controlled release of the drug in the avian digestive system [49]. Most birds consumed both uncoated and coated IVM feeds at similar rates to controls, although zebra finches consumed more coated IVM feed than either uncoated or control. Interestingly, this did not result in an increased IVM concentration in sera—rather, zebra finches fed coated IVM exhibited lower concentrations than those fed uncoated IVM. This could be attributed to Opadry® II suppressing the maximum concentration (*C*_max_), delaying *C*_max_ to peak later over the course of exposure, and extending the period of bioavailability. Birds were fed for a limited time period to minimize adverse metabolic effects induced by consuming a high-fat all-seed diet for several consecutive days [63]. Additionally, we did not assess the impact of IVM-formulated diets on different developmental stages of these avian species, as we do not anticipate songbirds, which primarily consume insects during the breeding season, to feed treated seeds to their young [64–67]. However, it is possible for birds to engorge themselves at a feeder if alternative sources of food are not available, so it will be critical to evaluate IVM-formulated feed for extended periods and at different ages. Beyond clinical observations and serum assessment, we selected liver tissue for histopathological examination in house sparrows because of the liver's role in avian IVM metabolism and the likelihood of detecting toxic changes shortly after exposure [50]. While we did not observe changes due to IVM exposure in this tissue after a 7-day diet, it is possible that other organs could accrue toxic changes because of IVM accumulation over time, and future work will incorporate histopathological assessment of many different tissues after extended diet periods. Conventionally, therapeutic doses of IVM in companion birds, poultry, and captive raptors range from 0.2 to 2.0 mg IVM/kg body weight [51, 52], with severe toxicity observed in chickens at 15 mg IVM/kg body weight delivered subcutaneously [53]. IVM selectively binds to invertebrate glutamate-gated chloride channels, but at high enough concentrations, it can potentiate the structurally similar vertebrate γ-aminobutyric acid (GABA_A_)-gated chloride channels that inhibit interneurons in the central nervous system (CNS). To access these (GABA_A_)-gated chloride channels, IVM could pass beyond the blood-brain barrier by oversaturating P-glycoprotein transporters that limit drug uptake into the brain [54]. To prevent exceeding this limit of tolerance, future studies should characterize pharmacokinetic profiles of IVM metabolism over time and throughout different tissues in a variety of avian species. Overall, even though our extrapolated real doses were higher than previously documented toxic doses, IVM-treated birdseed was safe up to 100 mg/kg coated feed, with no clinical or histological signs of toxicity in any of the birds evaluated at this concentration. Importantly, in our experiments, birds ate only IVM-treated feed for 7 consecutive days, whereas in field trials, we would expect wild birds to supplement their diet with non-treated feed. Therefore, since we did not observe adverse effects in our laboratory birds at the target dose of 100 mg/kg, we do not expect to observe them in the field.

Colonized *Cx. tarsalis* were susceptible to sera from IVM-treated birds at all diet concentrations; however, field-collected mosquitoes had higher rates of survival and may be less susceptible. Interestingly, we saw no relationship between mosquitocidal effect and type of seed (e.g. millet, sunflower seed), IVM formulation (e.g. coated, uncoated), or sera IVM concentration, demonstrating that the excipient does not inhibit the ability of the treated bird’s sera from affecting mosquito survival. We do not expect field-collected *Cx. tarsalis* to have developed targeted resistance to IVM, and their higher survival may be due to the greater fitness of wild mosquitoes compared to highly inbred, genetically restricted laboratory mosquitoes such as ours [55]. Because most of the data presented in this study were generated from studies conducted with colonized mosquitoes, future experiments should determine survivorship in genetically diverse, field-collected mosquitoes to evaluate susceptibility in wild mosquito populations. Studies in other mosquito genera, including *Culex* and *Anopheles*, have specifically implicated ATP binding cassette transporters and cytochrome P450 monooxygenases (CYP P450) in IVM metabolism [56–58]. Mutations in several different detoxification enzymes, including CYP P450, hydrolases, and glutathione S-transferases (GSTs), have been identified in field-collected insecticide-resistant populations in areas with pyrethroid application [59], so it is possible that field-collected *Cx. tarsalis* from Northern Colorado may have a broad assortment of alleles that could contribute to functional resistance. However, we observed that both field-collected and colonized *Cx. tarsalis* typically died within the first 3 days after exposure to IVM in avian blood meals, and most IVM-exposed mosquitoes exhibited some degree of flaccid paralysis before recovery. These mosquitoes would be expected to die under typical competitive field conditions, suggesting greater efficacy than what we observed in field-collected *Cx. tarsalis* under laboratory conditions. We also observed lower mortality in mosquitoes fed blood from chickens fed IVM-treated feed beyond 1.5 weeks, but this coincided with rapid weight gain as expected in young birds and could have been due to an increase in body size, which decreased the effective dose of IVM. Overall, we observed mosquitocidal efficacy in *Cx. tarsalis* indirectly and directly fed sera from birds fed IVM-treated feed, with no correlation among real dose, IVM concentration, and mosquito survivorship. In vivo blood-feeding data show that avian IVM concentrations needed to kill most *Cx. tarsalis* are significantly less than what we measured in in vitro serum-replacement membrane feeding experiments (LC_50_ 66.03 ng/ml) [30]. While it is surprising to see a lack of clear dose-response between IVM and mosquitocidal effect, other groups have shown that IVM metabolites identified in human blood and produced in vitro can reduce survival in *Anopheles* mosquitoes, potentially contributing to a “post-ivermectin effect” observed in mass drug administration trials for malaria control [60–62]. It is possible that mosquito survivorship could be impacted by IVM metabolites specific to avian processing, which we were unable to detect using our targeted analytic method. In future studies, we could expand our techniques to include the identification and assessment of such metabolites in multiple species.

## Conclusions

The results of this study show that 100 mg/kg IVM-treated bird feed is safe for consumption by small passerines and mosquitocidal to blood-feeding *Culex tarsalis*. Because we aim to achieve the minimum effective dose with the lowest probability of adverse events in wild birds, we plan to move forward with 100 mg/kg in future studies to evaluate the impact of IVM-treated feed on field-collected mosquitoes and additional small passerine species and to reduce WNV transmission in the field.

## Supplementary Information

Additional file 1: Supplementary Table 1. GPS locations of trap locations for collecting adult *Culex tarsalis* in Northern Colorado, summer 2023. Mosquitoes were captured in surveillance traps placed on volunteer household properties in Larimer, Weld, and Morgan counties in Northern Colorado. Supplementary Table 2. Blood meal host identification data. Data presented in the standardized format described in [68]. Supplementary Table 3. Primer names and sequence information for blood meal host identification. Supplementary Table 4. GPS locations and characteristics of mosquito traps used to collect adult *Cx. tarsalis* for live feed on IVM-treated chickens. Mosquitoes were captured in surveillance traps placed northeast of Fort Collins, CO, primarily around the Prospect Ponds Natural Area and close to water-filled irrigation ditches. Supplementary Table 5. Histopathological scoring for house sparrows fed 100 mg/kg IVM bird feed. Supplementary Table 6. Real dose of IVM per bird per formulation. Dataset with average consumption per bird group, average weight, and calculated real dose per day of diet. Supplementary Table 7. IVM concentration in serum compared to survivorship and real dose. Dataset with IVM concentration per individual bird compared to mosquito bioassay data and calculated real dose. Supplementary Table 8. Spearman correlation for IVM serum concentrations vs. survivorship per formulation and bird species.

Additional file 2: Supplementary Figure 1. Colonized *Culex tarsalis* experience similar mortality when fed pigeon sera collected at different time points throughout experimental diet. Colony *Cx. tarsalis* were fed pigeon sera from birds fed IVM-formulated uncoated millet diet or excipient-coated millet diet, and mortality was measured over 7 days. Log-rank test, not significant in both comparisons.

## Data Availability

Data supporting the conclusions of the present study are included within the article. Data used and/or analyzed during this study are available from the corresponding author upon reasonable request.
