# Supplementary material for: Evaluation of the safety and insecticidal efficacy of ivermectin-treated bird feed formulations in different avian species
Source: Parasit Vectors. 2026 Mar 27;19:202. doi: 10.1186/s13071-026-07311-6 (PMC13151197; doi:10.1186/s13071-026-07311-6)
Supplement: Supplementary file 2 — Additional file 2: Supplementary Figure 1. Colonized Culex tarsalis experience similar mortality when fed pigeon sera collected at different time points throughout experimental diet. Colony Cx. tarsalis were fed pigeon sera from birds fed IVM-formulated uncoated millet diet or excipient-coated millet diet, and mortality was measured over 7 days. Log-rank test, not significant in both comparisons. [file 13071_2026_7311_MOESM2_ESM.docx]

**Supplementary information.**


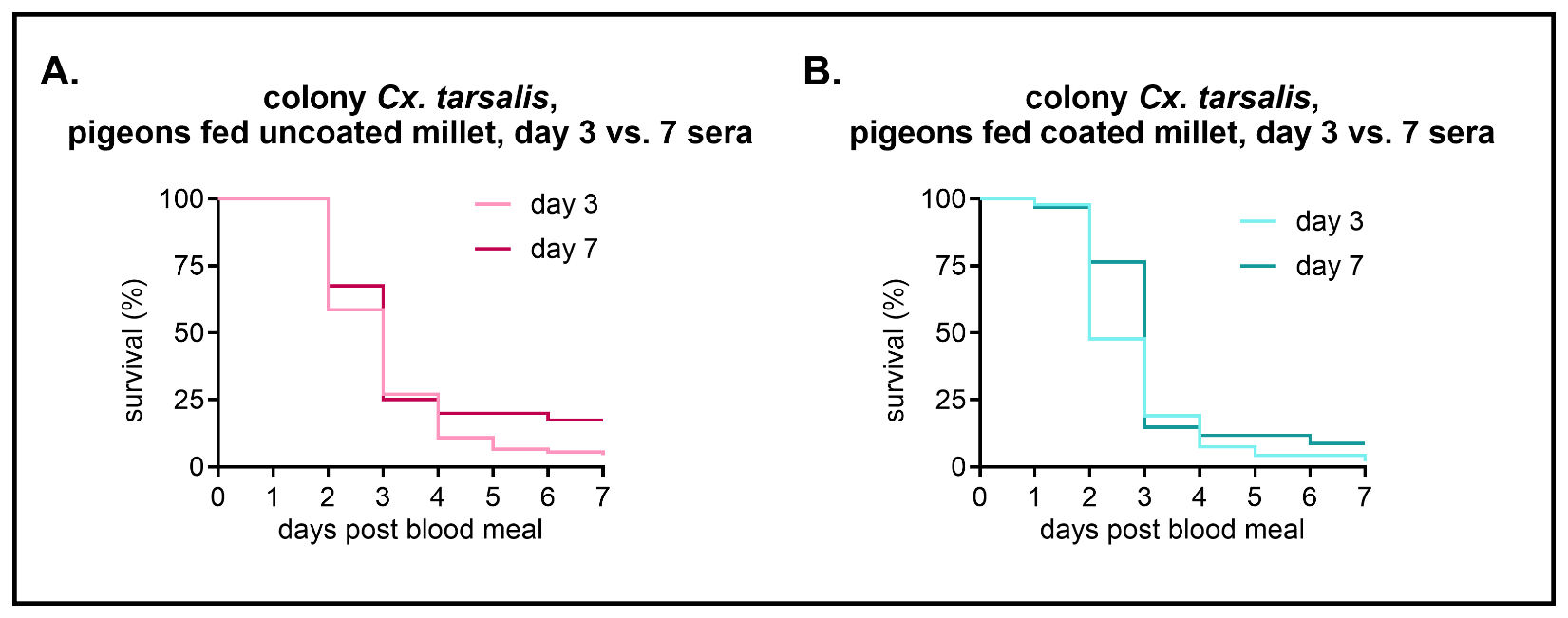


**Supplementary Figure 1. Colonized *Cx. tarsalis* experience similar mortality when fed pigeon sera collected at different time points throughout experimental diet.** Colony Cx. tarsalis were fed pigeon sera from birds fed IVM-formulated A) uncoated millet diet or F) excipient-coated millet diet, and mortality measured over seven days. Log-rank (Mantel-cox) test, not significant in both comparisons.
